# Protective Effect of Pitao (*Pitavia punctata* (R. & P.) Molina) Polyphenols against the Red Blood Cells Lipoperoxidation and the* In Vitro* LDL Oxidation

**DOI:** 10.1155/2018/1049234

**Published:** 2018-07-30

**Authors:** Ricardo I. Castro, Oscar Forero-Doria, Luis Soto-Cerda, A. Peña-Neira, Luis Guzmán

**Affiliations:** ^1^Multidisciplinary Agroindustry Research Laboratory, Universidad Autónoma de Chile, Talca, Chile; ^2^Carrera de Ingeniería en Construcción e Instituto de Ciencias Biomédicas, Universidad Autónoma de Chile, Talca, Chile; ^3^Instituto de Química de Recursos Naturales, Universidad de Talca, Talca, Chile; ^4^Facultad de Ciencias Forestales, Universidad de Talca, Talca, Chile; ^5^Centro de Plantas Nativas de Chile (CENATIV), Universidad de Talca, Talca, Chile; ^6^Department of Agro-Industry and Enology, Faculty of Agronomical Sciences, University of Chile, P.O. Box 1004, Santiago, Chile; ^7^Departamento de Bioquímica Clínica e Inmunohematología, Facultad de Ciencias de la Salud, Universidad de Talca, Talca, Chile

## Abstract

The oxidative stress is characterized by an imbalance between the oxidizing agents and antioxidants; meanwhile, the consumption of antioxidants has been considered as an important tool in the prevention of oxidative stress and its consequences.* Pitavia punctata* (R. & P.) Molina is an endemic arboreal species from the Chilean Coast Range, in which a large amount of flavonoids has been described. This work focused on characterizing and evaluating, in human erythrocytes, the antioxidant capacity and membrane protection of* P. punctata* extracts and the* in vitro* protection of the oxidation of the Low Density Lipoprotein (LDL). The phytochemical screening revealed the presence of Quercetin derivatives and flavonoids, such as (-)-Epicatechin, Kaempferol, and derivatives. The methanolic extract presented an important antioxidant activity, protecting the membrane integrity of the red blood cells against the oxidative damage caused by Hypochlorous acid and inhibiting the oxidation of the LDL lipoprotein.

## 1. Introduction

Nowadays, one of the major public-health problems is the metabolic syndrome (MS), which increases the risk of cardiovascular diseases and type II diabetes [1]. Many of the complications of the MS have been strongly associated with oxidative stress, characterized by an imbalance between the oxidizing agents (reactive oxygen species, lipid peroxidation) and antioxidants [2, 3].

Polyphenols have the capacity of scavenging free radicals and reactive oxygen species (ROS) [4, 5]. On the other hand, the flavonoids inhibit the nitric oxide synthase that generates nitric oxide, which reacts with free radicals to generate peroxynitrite species [6, 7]. The antioxidant mechanisms of phenols and flavonoids have not been yet elucidated; nevertheless, these compounds have the ability of partition in cell membranes, resulting in a restriction on the membrane fluidity, preventing the diffusion of free radicals [8].

**Figure 1:**
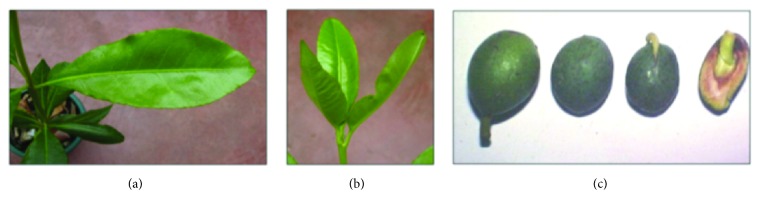
*Pitavia punctata*. (a) Leave; (b) whorled phyllotaxy; (c) fruits (photography taken from Dr. Alejandro Troncoso Aguilar records).

Many natural herbs with medicinal properties have been described [9]; among them,* Pitavia punctata *(R. & P.) Molina (Figure 1), an endemic arboreal species from the Chilean Coast Range in Central Chile (35°21′S – 37°41′S), belongs to a monotypic genus from* Rutaceae *[10], also known as “*Pitao*”, “*Canelillo*”, and “*Pitrán*” [11, 12]. In addition, a large amount of polyphenols has been described, and antioxidant activity in species of the family* Rutaceae*, e.g., citrus fruits, has large amounts of catechins, flavanones, and flavanonols, such as naringenin, hesperetin, liquiritin, and their glycosides [13], compounds with many antioxidant properties and low toxicities [14, 15].

Given the importance of antioxidants in the prevention of the MS and its consequences, the antioxidant capacity and membrane protection of* P. punctata* extracts and the* in vitro* protection of the oxidation of the Low Density Lipoprotein (LDL) were evaluated in human erythrocytes.

## 2. Materials and Methods

### 2.1. Chemicals

Caffeic acid, (−)-Epicatechin, Quercetin, Myricetin, Kaempferol Quercetin (-)-Epicatechin-3-*O*-gallate (E-3893) were purchased from Sigma Chemical Co. (St Louis, MO, USA). The flavonols Quercetin glucoside, Myricetin glucoside, and flavones Apigenin glucoside were purchased from Extrasynthèse (Genay, France). Flavonols Myricetin-3-*O*-galactoside, Myricetin-3-*O*-glucoside, Isorhamnetin-3-*O*-galactoside, Quercetin-3-galactoside, Quercetin-3-glucuronide, Quercetin-3-glucoside, Quercetin-3-rhamnoside, Kaempferol-3-galactoside, and Kaempferol-3-glucoside were provided by Apin Chemicals (Abingdon, Oxford, UK). All the solvents, (HPLC) grade, acetonitrile, acetic acid, formic acid, and methanol, were purchased from Merck (Darmstadt, Germany).

### 2.2. Plant Material and Extraction

Samples were collected in Cauquenes province, in “El Avellano, El Aromo” property (35°51′44′′S – 72°25′53′′O). This property is located next to Cauquenes-Pelluhue M-50 route and is surrounded by commercial plantations of* Pinus radiata* and* Eucalyptus *spp. This site presents a* Nothofagus glauca* forest, southwest exposure, and an elevation of 407 meters above sea level. The collection date was July 25th, 2013.* Pitavia punctata* botanical material was taxonomically determined by Luis Soto-Cerda, who has worked during the last ten years in coastal range forest research in the region of Maule. Later, this identification was corroborated by Alicia Marticorena, M.S., curator of the University of Concepción Herbarium. All samples were dried at room temperature (between 20 and 30°C) for three weeks in dark conditions. A voucher sample was stored in University of Concepción Herbarium (Voucher N° 176999), under controlled ambient conditions, with the Herbarium collection.

A methanolic extract was obtained from 310 g of dried plant in 1 L of methanol and then was filtered and taken to dryness under reduced pressure; a green color residue was obtained (16.6 g). The crude methanol sample (15.9 g of 16.6 g) was extracted successively with dichloromethane (DCM), acetone (Ac), and methanol (MeOH) at room temperature, respectively. The w/w yields in terms of dry starting material for the DCM, Ac, and MeOH extract were 10.0% (1.6 g), 1.80% (0.3 g), and 88.2% (14.0 g), respectively. All the extracts were tested for total phenols, flavonoid content, and free radical scavenging (DPPH).

### 2.3. Total Phenolic and Flavonoid Contents

The total phenolic contents of the extracts of* Pitavia punctata* were determined using the method described previously [16]. Briefly, a sample (160 *μ*L) of the different extracts was mixed with 100 *μ*L of Folin-Ciocalteu reagent and incubated 5 min before the addition of 300 *μ*L of 20% sodium carbonate (Na_2_CO_3_); the reaction tubes were diluted to 1.2 mL with distiller water and then incubated for 1 h. The absorbance of the mixture was read at 765 nm using a spectrophotometer (Thermo Spectronic GENESYS 10 UV) and the quantification was made on the basis of a standard curve of Gallic acid. The results were expressed as mg of Gallic acid equivalents (GAE) per gram of extract.

The total flavonoids contents of the extracts were determined by the methodology described previously [17]. Briefly, a sample (200 *μ*L) of the different extracts was mixed with 60 *μ*L of 5% sodium nitrite (NaNO_2_); after 6 min of incubation, 60 *μ*L of 10% aluminium chloride (AlCl_3_) was added and incubated for 6 min before the addition of 400 *μ*L of 4% sodium hydroxide (NaOH). As reference, a calibration curve was made using Quercetin as a standard; the absorbance of the reaction mixture was measured at 415 nm and the results were expressed as mg of Quercetin equivalents (QE) per gram of extract.

### 2.4. Antioxidant Activity

#### 2.4.1. DPPH (1,1-Diphenyl-2-Picrylhydrazyl) Radical Scavenging Method

The assay was done according to Brand-Williams et al., 1995 [18], adapted to microwells. Briefly, 75 *μ*L of several dilutions (1/2) of the extracts and control (80% methanol) was mixed with 150 *μ*L of DPPH reagent (20 mg/L in methanol). After 15 min of incubation, the absorbance was measured at 515 nm using a microplate reader spectrophotometer. The antioxidant activity (as percentage) was calculated as follows:

(1) $$\begin{array}{c} \%\text{ Scavenging DPPH free radical}=100\times \left(\;1-\frac{AS}{AD}\;\right) \end{array}$$

where AS is the absorbance of the extract dilution plus DPPH reagent and AD is the absorbance of the DPPH solution (20 mg/L). The half-maximal inhibitory concentration (IC_50_, mg/mL) was calculated by linear regression analysis and expressed as mean of three determinations. Quercetin was used as reference compounds.

#### 2.4.2. FRAP (Ferric Reducing Antioxidant Power) Assay

The FRAP reagent was prepared with 2.5 mL of 2,4,6-Tris (2-pyridyl)-s-triazine dissolved in 40 mM HCl, 25 mL of 300 mM of acetate buffer pH 3.6, and 2.5 mL of 20 mM FeCl_3_*∗*6  H_2_O [19]. Briefly, a sample (50 *μ*L) of the different extracts or standard (FeSO_4_*∗*7  H_2_O, Fe^2+^ concentration versus absorbance) was mixed with 1.5 mL of FRAP solution and diluted to a final volume of 1.7 mL with water. The reaction tubes were incubated at room temperature for 15 minutes and the product (ferrous tripyridyltriazine complex) was read at 593 nm, using a spectrophotometer (Thermo Spectronic GENESYS 10 UV). The percentage of Fe^3+^ scavenging (reduction to Fe^2+^) was calculated by comparison with the standard curve (*μ*mol Fe^2+^ per g of extract).

### 2.5. HPLC-DAD Analysis of Low-Molecular-Weight Phenolic Compounds

Several low-molecular-weight phenolic compounds were weighed (1.5 mg) and dissolved in 1 mL of 1:1 (v/v) methanol/water and membrane-filtered (0.45 *μ*m pore size) with an HPLC coupled to a diode array detector (Agilent ChemStation, 1200, USA), equipped with a low-pressure quaternary pump (model Agilent 1200) and autosampler (model Agilent 1260 Infinity Autosampler). The separation was made at 20°C using a Nova-Pak C18 column at 300 mm and the injection volume was 25 *μ*L of sample. All the phenols were determined by a direct readout of the sample absorbance at 280 nm. The photodiode array detector was set from 210 to 360 nm. Two mobile phases were used: A, water/acetic acid (98:2 v/v); B, water/acetonitrile/acetic acid (78:20:2 v/v/v), with a flow rate of 1 mL min^−1^ from 0 to 55 min and 1.2 mL min^−1^ from 55 to 85 min as follows: 100–20% A from 0 to 55 min, 20–10% A from 55 to 57 min, and 10–0% A from 57 to 85 min. Each major peak in the HPLC chromatograms was characterized by both the retention time and the absorption spectrum (from 210 to 360 nm).

### 2.6. Membrane Integrity of the Red Blood Cells

The membrane integrity of the red blood cells (RBCs) was determined according to Suwalsky et al., 2007 [20]. Briefly, RBCs were obtained from healthy volunteers by puncturing the ear lobule (blood sample 0.1 mL) in 10 *μ*L of heparin (5000 UI/mL). The RBCs were washed (three times) by adding 900 *μ*L of phosphate-buffered saline (PBS) pH 7.4 and, after gently mixed, were centrifuged at 1000 rpm × 10 min; the supernatant was discarded and replaced by the same volume of saline PBS pH 7.4. The washed RBCs (2%, v/v) were incubated with the selected extract of* P. punctata* (10 and 100 *μ*g/mL) for 15 min, then Hypochlorous acid (HClO) was added (25 *μ*M, final volume), and the solution was incubated for 1 h at 37°C, as a negative control 0.9% of sodium chloride (NaCl). After the incubation the samples were centrifuged (1000 rpm × 10 min) and the supernatant was discarded. The remaining RBCs were fixated by addition of 500 *μ*l of glutaraldehyde (2.5%, v/v) and examined by scanning electron microscopy.

### 2.7. Inhibition of RBCs Lipoperoxidation

The inhibition of the RBCs lipoperoxidation was carried out according to Yang et al., 2007 [21], with some modifications. Briefly, washed RBCs (100 *μ*L) at 50% were mixed with 20 *μ*L of different concentrations of the MeOH, Ac, and DCM extracts and 780 *μ*L of saline solution; the mixture was incubated for 10 min at 37°C. Then, 100 *μ*L of 1 mM* tert*-butylhydroperoxide (t-BHP) was added (under constant shaking) and, after 15 min of incubation at 37°C, 100 *μ*L of 1% Triton X-100 was added to stop the lipoperoxidation.

The lipoperoxidation process was measured by the thiobarbituric acid reactive substances (TBARS) assay. Briefly, 2 mL of thiobarbituric acid/trichloroacetic acid/hydrochloric acid (TBA/TCA/HCl) was added to reaction tubes along with the samples. The mixture was centrifuged at 3500 rpm for 5 min and the supernatant (2 mL) was boiled at 90°C for 15 min. The absorbance was read at 535 nm in a spectrophotometer (Thermo Spectronic GENESYS 10 UV). The half-maximal inhibitory concentration (IC_50_, *μ*g/mL) was calculated by linear regression analysis and expressed as mean of three determinations; as reference, a calibration curve was made using Malondialdehyde as a standard.

### 2.8. Isolation and Oxidation of Low Density Lipoprotein (LDL)

LDL isolated from plasma of healthy volunteers was obtained by differential centrifugation as described previously [22]. The isolated LDL (dialyzed with Ca^+2^–Mg^+2^-free PBS) was stored in dark at 4°C and the oxidation was prevented adding 45 mM BHT and 2 mM EDTA.

The production of conjugated dienes (LDL oxidation) was followed at 243 nm. For the oxidation, 50 *μ*g/mL of native LDL was mixed with cooper (5 *μ*M of CuSO_4_) in the absence (control) or presence of the extract (5 and 10 *μ*g/ml) dissolved in 0.9% NaCl; the generation of conjugated dienes was followed for 6 h, in a controlled temperature (37°C). The lag time was determined using the software GEN 5Ò by Biotek instruments. The results were expressed as means ± standard error (*n* = 6) and ascorbic acid (0.5 *μ*M) was used as a reference antioxidant.

### 2.9. Statistical Analysis

The results were expressed as means ± standard deviation of triplicate analyses. Data were statistically analyzed using the SPSS statistical software, version 15 (SPSS Inc., Chicago, IL). ANOVA test was used for comparison of means between groups with a level of significance of *p* < 0.05.

## 3. Results and Discussion

### 3.1. Phenol and Flavonoid Content

The concentrations of phenols obtained from the different extract were 130.8 ± 6.2 (DCM), 56.8 ± 1.3 (Ac), and 170.3 ± 8.6 (MeOH) mg GAE/g of extract, and for flavonoids they were 111.6 ± 1.2 (DCM), 17.6 ± 3.6 (Ac), and 137.5 ± 5.9 (MeOH) mg QE/g of extract. The phenolic and flavonoid content of these extracts diminished in a decreasing polarity order: MeOH > DCM > Ac, demonstrating a major concentration of both metabolites in the MeOH extract (Table 1).

### 3.2. Antioxidant Capacity of the* P. punctata* Extracts

The antioxidant capacity of the different extracts of* P. punctata* was tested by the free radical scavenging assay (DPPH); the results showed IC_50_ (mg/mL) values of > 0.1 (Ac), 0.098 ± 0.002 (DCM), and 0.07 ± 0.002 (MeOH). The radical scavenging effects of these extracts decreased in the polarity: MeOH > DCM > Ac, demonstrating that the most active extract was the MeOH extract (Table 1). For its part, the methanolic extract showed the higher capacity to reduce the Fe^3+^ to Fe^2+^ with 851.9 ± 7.4 *μ*mol Fe^2+^/g of extract, followed by the dichloromethane extract (548.2 ± 14.8 *μ*mol Fe^2+^/g of extract) and the acetone extract (185.2 ± 7.4 *μ*mol Fe^2+^/g of extract).

The results show a correlation between the flavonoid and phenolic content with the antioxidant activity. The presence of organic acids and polyphenols has demonstrated multiple biological properties* in vitro* and* in vivo* including antioxidant activities, among the phenolic antioxidants, with the flavonoid family being the most important class [23].

There is only one report about phytochemical study to the endemic Chilean specie* P. punctata* belonging to the* Pitavia* genus. In this report Silva et al., 1971 [24], isolated and characterized from leaves different secondary metabolites such as the furoquinoline alkaloids dictamnine, skimmianine and *γ*-fagarine in 0.004%, 0.0005%, and small quantities, respectively, based on dry plant weight. In addition different furoquinoline alkaloids have also been isolated from other members of the* Rutaceae* family. Thus, Rosas et al., 2011 [25], reported the presence of the furoquinolines maculosidine, robustine, and evolitrine among others, from the* Raputia praetermissa* species. Also, from the* Zanthoxylum buesgenii* species, three known furoquinolines have been reported: maculine, kokusaginine, and teclearverdoornine [26]. Likewise, from the* Helietta apiculata *[27] and* Sarcomelicope follicularis* [28] species, the presence of these alkaloids has been reported. On the other hand, from the benzene extract derived from leaves of* P. punctata* the common sterols *β*-sitosterol and daucosterin have been isolated, and in the methanolic extract Quercetin and avicularin, among other flavonols, were reported.

Different reports of the presence of different phenolic compounds in species belonging to the* Rutaceae* family could be found. At this respect, the specie* Skimmia anquetilia* (*Rutaceae*) has shown a total phenolic concentration of 1.1 mg/g of aqueous extract of oil obtained from the leaf with a good correlation among the phenolic content and the antioxidant activity [29]; for its part, a better antioxidant capacity (DPPH) was found in a bark extract of* Melicope glabra* with an IC_50_ of 13.01 ± 0.01 *μ*g/mL; also, a Pearson's correlation between the total phenolic content and the antioxidant activity (DPPH), using various concentrations, was calculated in 0.620, being statistically significant (*p* < 0.01) [30].

### 3.3. HPLC-DAD Analysis of Low-Molecular-Weight Phenolic Compounds

**Figure 2:**
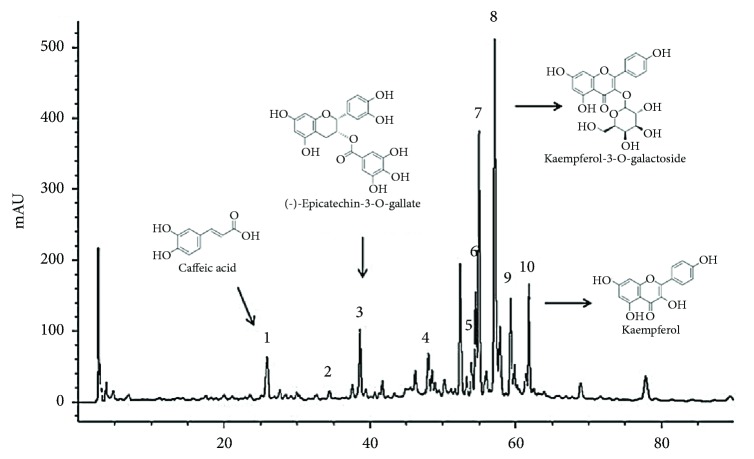
HPLC-DAD chromatograms of the MeOH extract of* Pitavia punctata*, detected at 280 nm. Peak numbers refer to Table 2.

The phenolic compounds identification from the MeOH extract of* P. punctata* was carried out by comparing the retention time and its absorption spectrum with those of the standard curve; in order to assign the identity of the peaks, each peak was labeled with a number according to its elution order in the chromatogram (Figure 2).

The phenolic compounds found in the MeOH extract include Quercetin derivates, previously described in* P. punctata* [24]; also, Quercetin derivates such as Quercetin-3-galactoside, Quercetin-3-glucuronide, and Quercetin-3-glucoside, were identified; additionally, other flavonoids such as (-)-Epicatechin, (-)-Epicatechin-3-*O*-gallate, Kaempferol, and derivates were identified; finally, phenols such as caffeic acid were identified (Table 2).

The presence of different phenolic compounds in the MeOH extract such as (-)-Epicatechin and its derivative (-)-Epicatechin-3-*O*-gallate and Quercetin derivates are some of the responsible for the antioxidant capacity of this extract, mainly for the characteristic presence of hydroxyl groups in the structure of these compounds that can donate hydrogen atoms in order to disrupt the chain propagation reaction of the oxidative process [31, 32]; moreover, neurological, antiviral, anticancer, cardiovascular, antimicrobial, and anti-inflammatory activities have been reported [33, 34]. The presence of Kaempferol flavonoid and its derivatives and the presence of caffeic acid (hydroxycinnamic acid) contribute to the antioxidant capacity thanks to its antioxidant power [35].

### 3.4. Morphology and Inhibition of RBCs Lipoperoxidation

**Figure 3:**
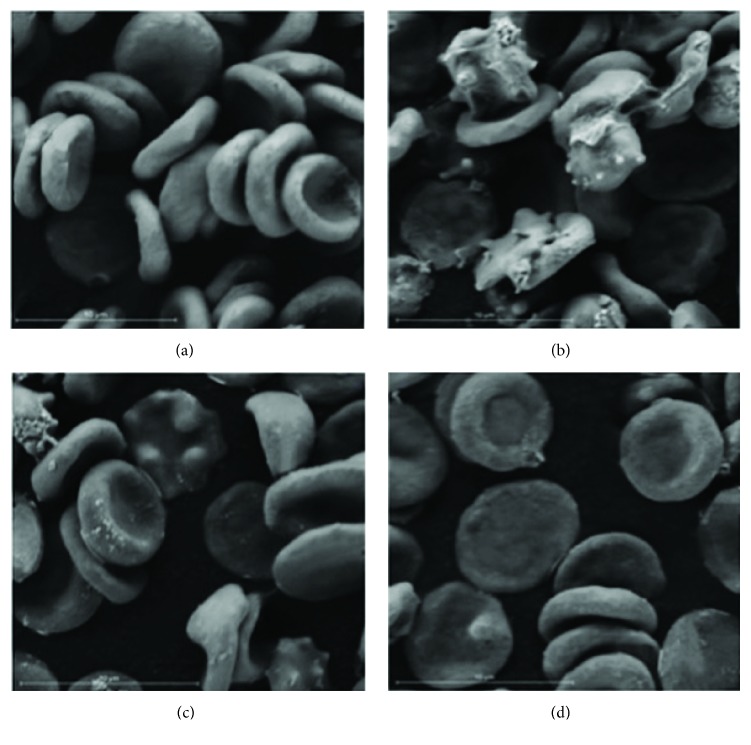
Morphology and membrane protection of RBCs by the MeOH extract of* P. punctata*. (a) Negative control (saline); (b) positive control HClO (25 *μ*M); (c) 10 *μ*g/mL of extract + HClO (25 *μ*M); (d) 100 *μ*g/mL of extract + HClO (25 *μ*M).

The morphology and membrane structure of the RBCs were studied by scanning electron microscope (SEM). After exposure of 25 *μ*M of HClO, the RBCs lose their normal shape showing changes in the morphology of the RBCs, mainly in the form of echinocytes (Figure 3(b)). The RBCs incubated with 10 *μ*g/mL of the methanolic extract of* P. punctata* and subjected to the oxidant agent HClO 25 *μ*M showed less changes compared with the positive control (HClO 25 *μ*M); meanwhile, the highest concentration used (100 *μ*g/mL) showed almost no changes in the morphology of the RBCs (Figure 3(d)), maintaining their biconcave discoid shape, demonstrating a membrane protection in a concentration dependent manner of the methanolic extract of* P. punctata*.

**Figure 4:**
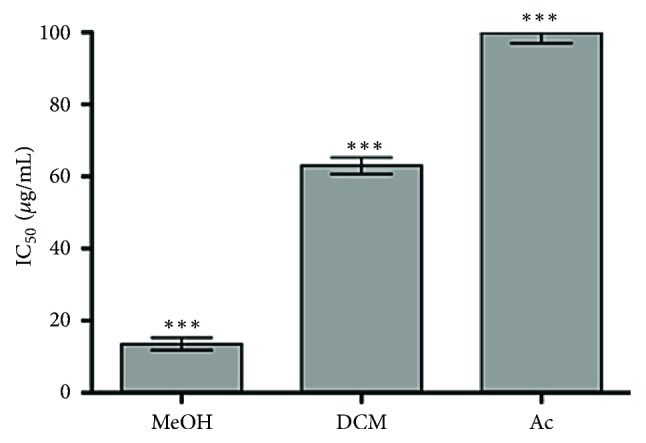
Inhibition of the lipoperoxidation (IC_50_) by the MeOH, DCM, and Ac extracts, induced by t-BHP. ^*∗∗∗*^*p* < 0.001 among them.

The lipoperoxidation analysis showed that the MeOH extract was the most effective, protecting the membrane of RBCs peroxidation with an IC_50_ of 13.6 ± 1.7 *μ*g/mL (Figure 4); the DCM extract showed poor protection against the lipoperoxidation with an IC_50_ of 63 ± 2.3 *μ*g/mL and the Ac extract showed no effect at the higher concentration used (IC_50_ > 100 *μ*g/mL).

The peroxidation of lipids has been accepted as one of the primary events in the oxidative cellular damage; at the same time, the RBCs are very susceptible to oxidation by various agents such as hydrogen peroxide, xanthine oxidase, dialuric acid, and organic hydroperoxides, inducing haemolysis and membrane damage [36].

The results showed that the MeOH extract of* Pitavia punctata* reduced the red blood cell damage induced by HOCl, in a concentration dependent manner; also, the MeOH extract was the most effective inhibiting the induced oxidation of lipids in the RBCs.* In vivo*, the strong oxidant HOCl is generated in substantial amounts via the myeloperoxidase-catalyzed oxidation of chloride by hydrogen peroxide [37, 38], and it has been demonstrated that the treatment of RBCs with HClO results in an inhibition of the Na^+^, K^+^, and Mg^2+^-ATPase activities; also, important oxidation of SH- groups, with changes in the membrane fluidity, surface area, and membrane morphological transformations that precede cell lysis, has been described [39, 40], thereby not only enabling a protective mechanism against the oxidation consequences but also preventing the endothelial dysfunction caused by potent oxidants such as HOCl, providing a vascular protection [41, 42]. In turn, it has been shown that polyphenols can not only act directly on the oxidizing agents, but also act in the lipophilic environment of the lipid bilayer. In this regard, Battino et al., 2012 [43], analyzed the phenolic extracts from two monofloral Cuban honeys for their total* in vitro* antioxidant capacity; their results showed the ability of the polyphenols to inhibit the oxidative damage and that this is likely to be due to their incorporation into cell membranes and their ability to cross it and reach the cytosol, confirmed by testing Quercetin (one of the most abundant flavonoids found in* P. punctata*), efficiently incorporated into erythrocytes.

### 3.5. Effect of the MeOH Extract of* Pitavia punctata* on Cu^2+^-Induced Human LDL Oxidation

**Figure 5:**
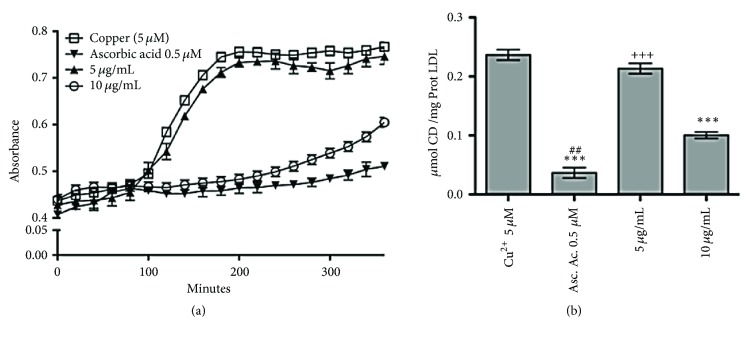
Effect of the MeOH extract of* P. punctata* on LDL oxidation. (**a**) Absorbance at 234 nm was measured every 2 min for 360 min at 37°C in a spectrophotometer to obtain a typical conjugated diene-formation (CD) curve. From the CD-formation curve, the lag time defined as end of the cross point of the time axis and the curve slope was estimated. (**b**) Conjugated dienes production at the final process of LDL oxidation. ^##^*p* < 0.001 with respect to 10 *μ*g/mL (MeOH extract); ^*∗∗∗*^*p* < 0.0001 with respect to Cu^2+^; ^+++^*p* < 0.0001 with respect to Asc. Ac. 0.5 *μ*M and 10 *μ*g/mL (MeOH extract).

The LDL incubated for 360 min with Cu^2+^ (5 *μ*M) was oxidized faster than the native LDL, with a lag time for the Cu^2+^-induced LDL oxidation of 78 ± 2.3 min; for its part, a concentration of 5 *μ*g/mL of the MeOH extract showed no statistically significant differences against the Cu^2+^-induced LDL oxidation (*p* > 0.05); nevertheless, a concentration of 10 *μ*g/mL of the MeOH extract showed a significant protection against the Cu^2+^ oxidation (Figure 5(a)); with a lag time of 271 ± 1.1 min (*p* < 0.001), the ascorbic acid 0.5 *μ*M completely inhibited the Cu^2+^-induced LDL oxidation. The quantification of the dienes produced at the end of the oxidation process showed a total amount of 0.238 *μ*mol CD/mg prot-LDL (Cu^2+^, 5 *μ*M), which was not different from the concentration of 5 *μ*g/mL of the MeOH extract (*p* > 0.05), but a concentration of 10 *μ*g/mL of the MeOH extract showed a reduction of 57.5% (0.101 *μ*mol CD/mg prot-LDL) in the CD formation induced by Cu^2+^ (Figure 5(b)), showing positive protection against the oxidation of this atherogenic lipoprotein.

The oxidative modification of the LDL is an independent risk factor for cardiovascular disease, playing an important role in the pathogenesis of atherosclerosis [44]. At a subendothelial level, the LDL suffers oxidation of their polyunsaturated fatty acid component due to the increased concentration of reactive oxygen species; these oxLDL particles promote the accumulation of lipid-laden foam cells in the intima with the further impairment of the endothelial function [45].

The inhibition of the LDL oxidation due to the antioxidant capacity of the MeOH extract of* P. punctata* could prevent the foam cells formation (accumulation of oxidized lipid within macrophages), which in turn could prevent the atherosclerotic process [46]; the HPLC-DAD chromatograms of the MeOH extract of* Pitavia punctata* showed that the main components of the polyphenols were Quercetin and Kaempferol. Quercetin has been extensively studied as a protective agent of the vascular health, showing important effects against the oxidation of LDL with hypocholesterolemic action [47, 48]; meanwhile, Kaempferol was the most abundant flavonoid in the MeOH extract; different studies have demonstrated the beneficial effect of this flavonoid on metabolic syndrome symptoms such as dyslipidemia [49, 50]; along with this, other studies have found a positive correlation between the consumption of Kaempferol or foods with an important content of Kaempferol with a reduced risk of type II diabetes and cardiovascular disease [51, 52].

## 4. Conclusion

Since the role of free radicals has been implicated in a large number of diseases, the antioxidant activity of different plants has a significant importance in exploiting their therapeutic potential. The present study showed the antioxidant property of different extracts of* Pitavia punctata*; among them, the MeOH extract shows the higher content of phenolic compounds and the best protective effect against the lipoperoxidation damage caused by HClO to the morphology and membrane structure of human erythrocytes, and it was also found to be an effective protection against the oxidation of an atherogenic lipoprotein as the LDL. In summary, the knowledge of the antioxidant potential present in natural renewable sources such as plants makes them a source of chemical diversity with high potential for treating various diseases caused by oxidative stress.

## Figures and Tables

**Table 1:** Total phenolic and flavonoid contents and antioxidant activity of the different extracts obtained from *P. punctata*.

Measurement	Methanol (MeOH)	Acetone (Ac)	Dichloromethane (DCM)	*p* value
Total Phenols (mg GAE/g of extract)	170.3 ± 8.6	56.8 ± 1.3	130.8 ± 6.2	*∗∗∗*
Total Flavonoid (mg QE/g of extract)	137.5 ± 5.9	17.6 ± 3.6	111.6 ± 1.2	*∗∗∗*
DPPH (IC_50_) (mg/mL)	0.07 ± 0.002	>0.1	0.098 ± 0.002	*∗∗*
FRAP (*µ*mol Fe^2+^/g of extract)	851.9 ± 7.4	185.2 ± 5.4	548.2 ± 6.2	*∗∗∗*

DPPH: 1,1-diphenyl-2-picrylhydrazyl; FRAP: *ferric reducing antioxidant power*. ^*∗∗*^*p* < 0.0018 among them; ^*∗∗∗*^*p* < 0.0001 among them.

**Table 2:** Tentative identification of phenolic compounds in the MeOH extract from leaves of *Pitavia punctata* by HPLC–DAD.

**Peak #**	**Rt (min)**	***λ*** **max (nm)**	**MW**	**Tentative identification**

1	25.8	280^s^, 323, 300^**s**^	178	Caffeic acid
2	34.4	280, 235	290	(−)-Epicatechin
3	38.6	280	442	(−)-Epicatechin-3-*O*-gallate
4	47.9	270, 340	-	Unknown
5	53.9	280^s^, 354, 300^**s**^, 256	464	Quercetin-3-galactoside
6	54.4	280^s^, 354, 300^**s**^, 256	478	Quercetin-3-glucuronide
7	55.9	280^s^, 354, 300^**s**^, 256	464	Quercetin-3-glucoside
8	57.1	280, 348	448	Kaempferol-3-*O*-galactoside
9	59.2	354, 256	-	Unknown
10	61.8	280^s^, 346, 256,	286	Kaempferol

^**s**^Shoulder

## Data Availability

The data used to support the findings of this study are included within the article.
